# Characteristics of Gut Microbiota in Rosacea Patients—A Cross-Sectional, Controlled Pilot Study

**DOI:** 10.3390/life14050585

**Published:** 2024-05-01

**Authors:** Anne Guertler, Pascal Hering, Cátia Pacífico, Nikolaus Gasche, Barbara Sladek, Miriam Irimi, Lars E. French, Benjamin M. Clanner-Engelshofen, Markus Reinholz

**Affiliations:** 1 Department of Dermatology and Allergy, LMU University Hospital Munich, 80337 Munich, Germanylars.french@med.uni-muenchen.de (L.E.F.);; 2 Biome Diagnostics GmbH, 1200 Vienna, Austria; 3 Dr. Phillip Frost Department of Dermatology and Cutaneous Surgery, Miller School of Medicine, University of Miami, Miami, FL 33136, USA

**Keywords:** gut–skin axis, stool, enterotypes, diversity, nutrition, diet

## Abstract

Background: Recent studies have suggested a possible connection between rosacea and patients’ gut microbiota. Objective: To investigate the differences in fecal microbial profiles between patients with rosacea and healthy controls. Methods: Gut microbiota of 54 rosacea patients (RP) were analyzed using MiSeq 16S rRNA sequencing. Enterotypes, the Firmicutes/Bacteroides (F/B) ratio, the significance of alpha and beta diversity, and differential abundance analysis (DAA) were calculated and compared with age- and gender-matched controls (CP, *n* = 50). Results: Significant changes in the enterotypes and F/B ratio were observed between the RP and CP (*p* = 0.017 and *p* = 0.002, respectively). The RP showed a decreased microbial richness and diversity compared to the CP (Shannon *p* = 0.012, inverse Simpson *p* = 0.034). Beta diversity also differed between both groups (PERMANOVA, *p* = 0.006). Fourteen significantly different taxa were detected according to DAA. *Faecalibacterium prausnitzii* (coef. −0.0800, *p* = 0.008), *Lachnoospiraceae ND 3007 group* sp. (coef. −0.073, *p* < 0.001), and *Ruminococcaceae* (coef. −0.072, *p* = 0.015) were significantly decreased; *Oscillobacter* sp. (coef. 0.023, *p* = 0.031), *Flavonifractor plautii* (coef. 0.011, *p* = 0.037), and *Ruminococccaceae UBA 1819* (coef. 0.010, *p* = 0.031) were significantly increased in the RP compared to the CP. Conclusion: Significant alterations in gut microbiota were present in the RP. Taxonomic shifts and reduced richness and diversity were observed when compared to the CP. Larger prospective studies are needed to investigate correlations with clinical features and to translate these findings into future therapeutic approaches.

## 1. Introduction

Once solely considered as a skin disorder manifesting with persistent or recurrent facial erythema, papules, and pustules, accompanied by the presence of phymatous lesions and possible ocular symptoms, rosacea is now acknowledged as a multisystem inflammatory disease [1,2,3]. With a global prevalence estimated at 5.5% of adults, particularly those with fair skin, affecting men and women equally, rosacea can lead to an impaired quality of life, stigmatization, and anxiety [4]. Although the exact pathophysiology of rosacea remains unclear, genetic factors, dysregulation of the innate and adaptive immune systems, abnormal neurovascular responses, and changes in the cutaneous microbiome have been implicated as driving forces [2]. As many patients also experience gastrointestinal (GI) symptoms, the concept of the so-called gut–skin axis has recently been proposed in rosacea, suggesting a possible link between GI and skin health [5]. Clinical experience suggests that GI symptoms in rosacea patients are often overlooked in the presence of facial symptoms, particularly if they are not actively addressed by clinicians [6]. However, comorbidities can range from abdominal pain to bloating, constipation, and diarrhea, with an increasing number of rosacea patients being diagnosed with *Helicobacter pylori* infection, inflammatory bowel disease (IBD), including Crohn’s disease and Colitis ulcerosa, and small intestine bacterial overgrowth (SIBO) [7,8]. While the skin microbiome in individuals with rosacea has been extensively studied in recent years, leading to therapeutic advances in prescription treatments, it is now crucial to investigate the gut microbiota of these patients [9].

The gut microbiota is a complex community of microorganisms, primarily bacteria, inhabiting the GI tract that has established a close symbiotic relationship with its human host [10]. Its integrity is influenced by demographic factors and genetics, but also by environmental factors, including diet [11]. Alterations may lead to a so-called dysbiosis, which involves the selection of specific microorganisms that potentially modulate the immune function and levels of inflammation and thereby affect the host’s health and disease course. Regarding rosacea, there are very few data on the gut microbiota composition in affected patients [5]. However, based on clinical studies of other inflammatory skin diseases, such as atopic dermatitis, psoriasis, or acne vulgaris, several mechanisms have been proposed to explain how an intestinal dysbiosis may affect skin inflammation [12,13,14]. For example, a dysbiosis may lead to a compromised intestinal mucosal barrier function with translocation of bacteria and their metabolites from the gut into the bloodstream and ultimately to the skin [15]. Intestinal dysbiosis can also lead to a general systemic inflammatory response, which can also exacerbate inflammatory skin conditions. In addition, neuroendocrine pathways have been described, through which the gut and skin microbiota may communicate [16].

A better understanding of the gut–skin axis also has future therapeutic implications. In particular, modulation of the gut microbiota is emerging as a promising target for the prevention and treatment of inflammatory skin diseases [17,18]. For example, targeted antimicrobial therapies can be used to combat an increase in potentially pathogenic species, while the administration of specific probiotics can address the loss of beneficial commensals [19]. Clinical trials have shown beneficial results in patients with atopic dermatitis [20], psoriasis [21], or acne vulgaris [22]. However, there is still a need for evidence regarding rosacea.

To fill this gap, this cross-sectional, controlled study uses 16s rRNA gene sequencing to investigate the composition of the gut microbiota in a Western rosacea cohort in comparison to healthy controls. Based on these data, targeted interventions, including probiotic supplementation aimed at modulating the gut microbiota, may shape the therapeutic future for rosacea.

## 2. Material and Methods

### 2.1. Study Design

This cross-sectional pilot study was conducted at the Department of Dermatology and Allergy of the Ludwig-Maximilians-Universität (LMU), Munich, Germany, between November 2021 and December 2022. The study included adult patients with clinically diagnosed rosacea, regardless of their current clinical presentation or treatment. Patients were recruited from the department’s specialized outpatient clinic for inflammatory facial dermatoses. Exclusion criteria were pregnancy and breastfeeding. Written informed consent was obtained from all the participants prior to their inclusion in the study. The study adhered to the tenets of the Declaration of Helsinki and received ethical approval from the Ethics Committee of the Faculty of Medicine (Ref.-No. 21-0932).

### 2.2. Assessments and Outcomes

#### 2.2.1. Stool Sample Collection

Stool samples were collected by the patients themselves using a home stool collection kit (Norgen Biotek Corp., Thorold, ON, Canada). The sampling tubes contained a preservative solution to stabilize the microbial composition at room temperature during transport. The samples were returned to a specialized laboratory (Biome Diagnostics GmbH, Vienna, Austria) and stored at −20 °C upon arrival until processed for amplicon sequencing.

#### 2.2.2. DNA Extraction and Sequencing

Microbial DNA was extracted on a KingFisher FLEX (Thermo Scientific, Waltham, MA, USA) using the innuPREP AniPath DNA/RNA Kit 2.0-KFFLX (IST Innuscreen, Berlin, Germany) according to the manufacturer’s instructions. DNA quality and quantity were assessed using a NanoDropTM 2000c spectrophotometer (Thermo Scientific) and fluorometrically using the Quant-it TM Pico Green TM dsDNA Assay Kit (Invitrogen by Thermo Scientific) on a SpectraMax M2 (Molecular Devices, San Jose, CA, USA). Amplicon sequencing was performed using the barcoded primers 341F (F-forward primer; 5′-CCTACGGGNGGCWGCAG-3′) and 806R (R-reverse primer; 5′-GGACTACHVGGGTATCTAATCC-3′) flanking the V3-V4 hypervariable region of the eubacterial 16S rRNA gene. Approximately 50–1000 ng of genomic DNA per sample was used for library construction. Sequencing was performed on an Illumina MiSeq platform using a 2 × 300 bp paired-end reads approach. The libraries were sequenced to a uniform high-depth target of 50,000 paired-end reads. The bacterial stool composition of the rosacea patients was compared to that of a control group of healthy, sex- and age-matched European individuals from the specialized laboratory’s database (individuals between 18 and 65 years of age, with no antibiotic use three months prior to the study and without a history of IBD or skin diseases).

#### 2.2.3. Bioinformatic Analysis

The raw reads were filtered and denoised using DADA2 v1.18.0, yielding high-quality sequences. The SILVA database v138 was used to perform a taxonomy assignment for each unique amplicon sequence variant (ASV). The samples were rarefied to 20,000 reads per sample. The ASV table, taxonomy, and metadata were imported into a TreeSummarizedExperiment using the mia package v1.3.23.

The bacterial taxa were classified into hierarchical levels based on phylum, class, order, family, and genus. The relative abundances of the top ten bacterial classes were displayed. The samples were categorized into enterotypes I, II, and III (I: high in Bacteroides and low in Prevotella (Bacteroides enterotype (B-type)); II: high in Prevotella and low in Bacteroides (Prevotella enterotype (P-type)); III: higher population of genus Ruminococcus within the phylum Firmicutes (Ruminococcaceae enterotype (R-type)) [23]. The ratio of Firmicutes/Bacteroidetes (F/B) was calculated [24].

Microbiome richness and diversity were evaluated through alpha diversity metrics, which included the number of observed ASVs as a measure of richness, as well as Shannon diversity and the inverse Simpson as measures of diversity. To measure the distance between samples, Jaccard and Aitchison distances were calculated on centered log-ratio (clr)-transformed values. Alpha diversity summarized the species abundances in a sample into a single number. For all the calculated alpha diversity measures, greater values indicated greater sample diversity. Additionally, beta diversity was used to quantify (dis)similarity between samples; the beta diversity measures included the Bray–Curtis index, Euclidean distance, and Aitchison distance (clr-transformed data).

#### 2.2.4. Clinical Severity

Rosacea predilection sites (face, chest, and eyes) were assessed according to the global ROSacea COnsensus panel (ROSCO) criteria, and the overall clinical severity was graded by an independent dermatologist using a 3-point scale (mild, moderate, and severe), enabling a subdivision of the rosacea cohort (mild vs. moderate/severe) [4]. Digital photography was used to ensure objective documentation of the patients’ skin condition using a Nikon D5 with an AF-S Nikkor 60 mm lens.

#### 2.2.5. Questionnaire Data

Rosacea patients were asked to complete a comprehensive study-specific questionnaire, which was developed by the investigators and used to collect detailed information on demographics, including a differentiation between urban and rural residence (>/<300 inhabitants/km^2^), occupation, current and past prescription rosacea treatments, supplement intake, alcohol or nicotine consumption, medical history with a special emphasis on recurring GI symptoms (defined as >3×/week), diagnosed GI comorbidities, and allergies, as well as information on exposome factors (stress level (0–10: none–constant), sleep (>/<7 h/daily), and daily physical activity (>/<30 min of walking/exercise). The participants were also asked to subjectively rate various foods based on whether they had a positive or negative effect on their clinical severity.

The Dermatology Life Quality Index (DLQI) was used as a standardized tool to assess the impact of rosacea on patients’ quality of life, including psychological disability at work, social and sexual relationships, depression, and anxiety [25]. The DLQI scores ranged from 0 to 30, with higher scores indicating a greater impact on quality of life (0–1: no impact; 2–5: small impact; 6–10: moderate impact; 11–20: very large impact; and 21–30: extremely large impact).

### 2.3. Primary Objective

The primary objective of this study was to investigate the characteristics of gut microbiota in rosacea patients compared to healthy controls.

### 2.4. Secondary Objectives

The secondary objectives were defined as follows:

- To rate the clinical severity of rosacea;
- To explore the participants’ quality of life;
- To offer clinicians a better understanding of microbiome data and to present clinical implications.

### 2.5. Statistical Evaluation

The analysis of the demographic data was conducted using the SPSS software version 26 (IBM, Armonk, NY, USA). The level of significance for all the statistical tests was set at a *p*-value of 0.05, ensuring robust and reliable results. Descriptive statistics, including mean, median, standard deviation, and minimum and maximum values, were calculated to summarize the demographic data. Appropriate parametric and non-parametric tests were selected and applied based on the nature of the data. Multivariate analyses were conducted to explore potential relationships between the variables and the outcomes of interest. Graphs were created using Microsoft PowerPoint (Version 16.54). Significance among the alpha diversity indices was tested using the Wilcoxon test. Permutational multivariate analysis of variance (PERMANOVA) was calculated in R v4.1.3 with the adonis2 function and used to assess significant beta diversity differences between groups. Differential abundance analysis (DAA) was used to detect differences in the abundances of individual taxa between rosacea and the control. MaAsLin2 v1.8.0. relies on a general linear model, whereas ALDEx2 v1.26.0 uses the Wilcoxon rank sum test and Welch’s *t*-test to infer abundance differences. For both methods, Benjamini–Hochberg corrected *p*-values were used, and the statistical significance was based on a 95% confidence interval.

## 3. Results

### 3.1. Patients’ Demographics

The study population comprised 54 rosacea patients (39 females and 15 males), with an average age of 45.3 ± 13.6 years; the patients predominantly lived in urban areas. A summary of their demographic characteristics is provided in Table 1.

Most of the patients presented with moderate to severe clinical severity; 79.6% of the total cohort (*n* = 43) were undergoing topical prescription treatment (Ivermectin (*n* = 37, 68.5%), Metronidazole (*n* = 27, 50%), Brimonidine (*n* = 2, 3.7%), and Azelaic Acid (*n* = 1, 1.9%)). Systemic treatment was reported by 24.1% (*n* = 13) of the patients (Doxycycline (*n* = 11, 20.4%), Isotretinoin (*n* = 1, 1.9%), and Metronidazole (*n* = 1, 1.9%)), most of whom suffered from moderate to severe clinical presentations compared to mild presentation (*p* = 0.009). Over half of the total cohort self-reported regularly recurring GI symptoms (*n* = 29, 53.7%), irrespective of the clinical rosacea severity, with confirmed diagnoses in six cases. The patients experienced an overall low impact of rosacea on their quality of life, demonstrated by a mean DLQI score of 5.9, with a significant difference between mild and moderate/severe clinical presentation (*p* = 0.041).

### 3.2. Relative Abundance, Enterotypes, and Firmicutes/Bacteroides (F/B) Ratio

**Figure 1:**
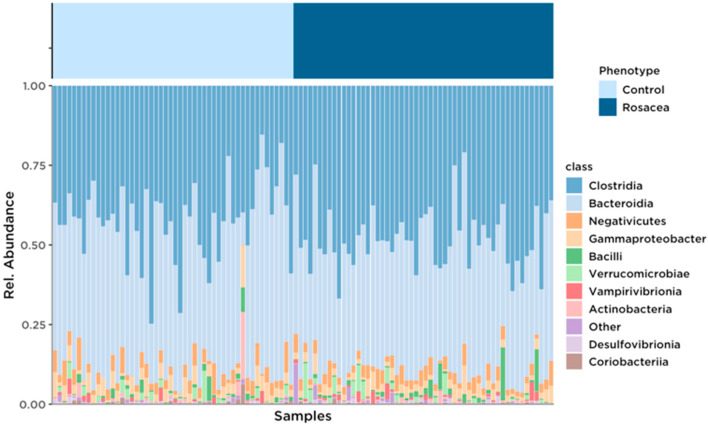
Bar plot with calculated relative abundances in rosacea patients and controls. Taxa with the highest abundances at the class level were visualized for each sample.

The relative abundance of the top ten bacterial classes of the intestinal microbiota is presented in Figure 1.

The fecal microbiota in both study groups was dominated by Clostridia (phylum Bacillota, 44.62%) and Bacteroidia (phylum Bacteroidota, 43.79%) at the class level, followed by Negativicutes, Gammaproteobacteria, Bacilli, Verrucomicrobiae, Vampirivibrionia, Actinobacteria, Desulfovibrionia, and Fusobacteriia (cumulatively 10.98%).

The differentiation of the enterotypes revealed significant differences between the rosacea and control subjects (*p* = 0.017). While enterotype I was most frequent in both groups (rosacea 57.4% (*n* = 31); control 48.0% (*n* = 24)), enterotype III was more frequent in the rosacea patients (27.8% (*n* = 15)) compared to the control patients (13.0% (*n* = 7)), whereas enterotype II was more frequent in the control patients (38.0% (*n* = 19)) compared to the rosacea patients (14.8% (*n* = 8)).

The comparison of the F/B ratio showed significant differences between the rosacea and control patients (*p* = 0.002). A decreased ratio was found in 28 rosacea patients (51.9%) and 10 control patients (20.0%), while an increased ratio was found in 26 rosacea subjects (48.1%) and 40 control subjects (80%).

### 3.3. Richness and Diversity

**Figure 2:**
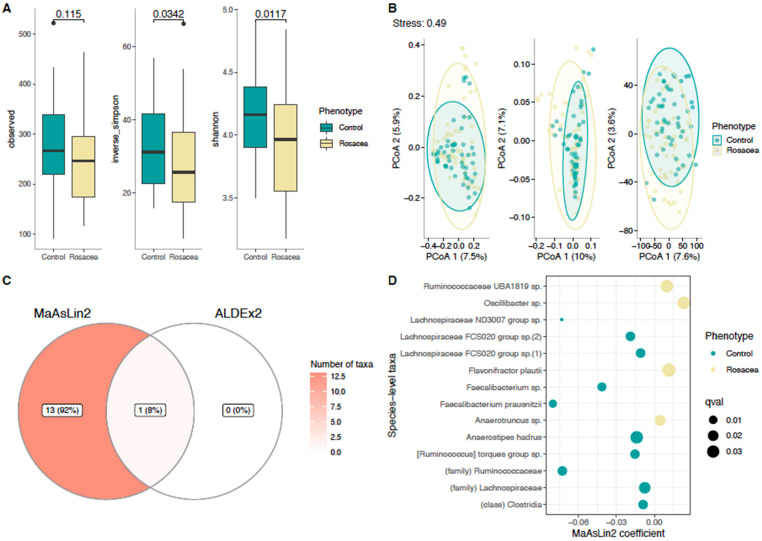
(**A**) Microbial richness (number of observed operational taxonomic units [ASVs] (left column)) and diversity measures (inverse Simpson (middle column), Shannon index (right column)) of intestinal microbiota between rosacea patients and controls. (**B**) Principal coordinates analysis (PCoA) plots are shown with the percentage of explained variance. In PCoA with non-Euclidean distances, dissimilarities are first projected into similarities in a Euclidean space (with some information loss, i.e., stress) and then projected to the maximal variance axes. This means that the maximal variance axes do not necessarily reflect the correspondence of the projected distances and original distances. Hence, the classical stress function (which sums up the squared differences and scales them to the squared sum of the original ones) is reported here along the PCoA plots. Stress varies between 0 and 1, and smaller stress values mean better scaling. (**C**) Differential abundance analysis (DAA) using ALDEx2 and MaAsLin2. Taxa were analyzed at species level. Only statistically significant associations are shown. (**D**) Detailed differential abundance analysis (DAA) using MaAsLin2 between rosacea patients and controls.

Significant differences were observed with regard to alpha diversity, with a reduced species abundance in the rosacea patients compared to the controls, according to both the Shannon diversity index (*p* = 0.012) and the inversed Simpson (*p* = 0.034, Figure 2A). Furthermore, rosacea and control cases differed in their beta diversity, with significant differences in the microbial community structure (Figure 2B, *p* = 0.006). The analysis of the confounding factors using PERMANOVA revealed a significant impact of gender (*p* = 0.042).

### 3.4. Differential Abundance Analysis of Taxa

The differential abundance analysis showed 14 significantly different taxa between the rosacea patients and the controls, with *Lachnospiraceae* ND 3007 detected by two independent methods, MaSsLin2 and ALDEx2 (Figure 2C). MaAsLin2 showed a reduced abundance of *Faecalibacterium prausnitzii* (*F. prausnitzii*) (coef. −0.0800, *p* = 0.008), *Lachnoospiraceae* ND 3007 group sp. (coef. −0.073, *p* < 0.001), *Ruminococcaceae* (coef. −0.072, *p* = 0.015), and *Faecalibacterium* sp. (coef. −0.04, *p* = 0.012) in the rosacea cohort compared to the controls. An increased abundance of *Oscillobacter* sp. (coef. 0.023, *p* = 0.031), *Flavonifractor plautii* (coef. 0.011, *p* = 0.037), *Ruminococccaceae* UBA 1819 (coef. 0.010, *p* = 0.031), and *Anaerotruncus* sp. (coef. 0.005, *p* = 0.021) was detected in the rosacea patients (Figure 2D).

No significant associations were found between the clinical severity of rosacea or the presence of GI symptoms and microbiota characteristics.

### 3.5. Dietary Assessment

**Figure 3:**
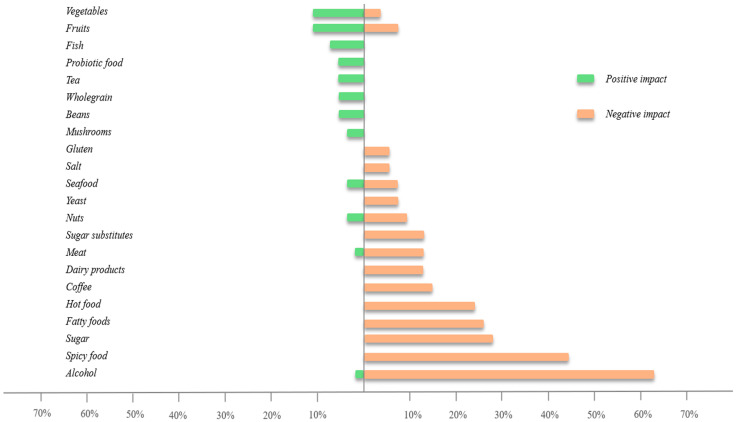
Rosacea patients’ subjective assessment (%) of food items with beneficial (green) and negative impact on the clinical severity (red).

Overall, more foods were identified as rosacea triggers than beneficial items (Figure 3). Alcohol was subjectively perceived as the main dietary trigger, followed by spices, refined sugar, fried/fatty foods, hot food, coffee, dairy, meat, and sugar substitutes. Vegetables, fruits, fish, probiotics, tea, wholegrain, and legumes were perceived as most favorable.

## 4. Discussion

Over the past decade, substantial evidence has highlighted the significant impact of the gut microbiota on overall immune response, modulation of inflammation, and more recently, skin health [16,26,27,28]. This pilot study, the largest to date and the first to be conducted in a Western cohort, identified significant differences in the gut microbiota of rosacea patients compared to healthy controls.

Neither the enterotype nor the F/B ratio has previously been studied in rosacea patients. Enterotypes are not simply an enumeration of bacteria, but rather a categorization of digestive functions; they are associated with long-term dietary habits, independent of gender and age [29,30,31]. Bacterial clusters of enterotype I, which were predominantly found in both study cohorts, have been described as being most prevalent in individuals from European countries who adhere to a Western diet characterized by a high intake of animal protein and saturated fat [29]. As the study cohort only included patients from Europe, this finding was not surprising. Interestingly, bacterial clusters of enterotype III are associated with a carbohydrate-based diet and were seen more frequently in the presented cohort of rosacea patients compared to the controls. Enterotype II clusters are associated with a more plant-based diet rich in fiber and were found to be least frequent in the rosacea patients but more frequent in the control group. Several associations between enterotypes and disease phenotypes have been reported in humans, but data for dermatological conditions are scarce [32]. Future studies are needed to investigate the clinical relevance of enterotypes in rosacea patients. In particular, an investigation is needed to determine whether a more plant-focused diet over several weeks would increase the number of rosacea patients with enterotype II and how this would affect their clinical severity.

The F/B ratio has been studied in relation to various health conditions [33]. Increased or decreased F/B ratios are regarded as dysbiosis; the former has been observed in obesity and type 2 diabetes, and the latter in patients with IBD [19,34]. The present analysis showed significant differences between the rosacea patients and the healthy controls. While the control participants predominantly presented with an increased ratio, the rosacea patients showed both alterations, neither of which resembled any previously described pattern. As no comparable data have yet been published, it is currently difficult to associate the F/B ratio with rosacea. However, we propose that the integration of the F/B ratio assessment into clinical models may be an interesting tool to better understand the role of GI symptoms in rosacea patients.

Comparable research on the gut microbiome with the assessment of the alpha and beta diversity measures of rosacea patients is currently limited to two exploratory studies from Asia [35,36]. Nam et al. conducted a cross-sectional study of 12 Korean women with rosacea along with 251 controls, whereas Chen et al. studied 11 rosacea patients in Taiwan (10 women) compared to 110 controls, with both studies examining the gut microbial richness and composition using 16S rRNA gene sequencing. Regarding the evaluation of alpha diversity measures, conflicting outcomes emerged from the studies. Nam et al. reported no significant difference between rosacea patients and healthy controls, whereas Chen et al. observed a significant decrease in the fecal microbiome when using observed OTUs for statistical analysis, but no significant difference when using the Shannon index. Our study revealed a decreased microbial diversity in the rosacea patients in comparison to the controls, as indicated by both the Shannon diversity index (*p* = 0.012) and inverse Simpson (*p* = 0.034). As a higher diversity is a marker of health [37], a reduced diversity in rosacea patients may be a possible anchor for treatment interventions. Attempts to promote microbiota diversity include dietary modification with fermented foods or probiotic supplements. However, there is a lack of clinical or preclinical evidence for oral supplementation in rosacea [5], but we hypothesize that oral supplementation would increase measures of alpha diversity.

Regarding beta diversity, both previous studies showed significant differences between rosacea patients and healthy volunteers, as did our study, which also showed a separation of the rosacea and control cases in beta diversity according to PERMANOVA (*p* = 0.006). Comparing the DAA results, Nam et al. found that the abundance of *Methanobrevibacter*, *Slackia*, *Coprobacillus*, *Citrobacter*, *Desulfovibrio*, and an unknown genus of the *Peptococcaceae* family were decreased in rosacea patients, while they observed increased levels of *Megasphaera*, *Acidaminococcus*, and *Lactobacillales* order unknown family unknown genus in the same population. In contrast, Chen et al. found increased levels of *Rhabdochlamydia*, *CF231*, *Bifidobacterium*, *Sarcina*, and *Ruminococcus* and decreased levels of *Lactobacillus*, *Megasphaera*, *Acidaminococcus*, *Hemophilus*, *Roseburia*, and *Clostridium*. As the microbiome profile is highly dependent on demographic characteristics, such as age and gender, our results in a Western cohort with a younger mean age (25 vs. 52 and 43 years) and a more balanced gender profile revealed completely different taxa between the rosacea patients and the controls compared to the results from the Asian cohorts. In our population, reduced abundances of *F. prausnitzii*, *Lachnospiraceae* ND 3007 group sp., and *Ruminococcaceae* were found compared to the healthy controls, with a significant increase in *Oscillobacter* sp., *Flavonifractor plautii*, *Ruminococccaceae* UBA 1819, and *Anaerotruncus* sp. While data on the role of these bacteria in dermatologic conditions are scarce, no clinical implications for rosacea patients have been described. 

*Ruminococcaceae* have been found to be increased in samples from IBD patients, which may represent another parallel to GI symptoms in rosacea patients [38]. *F. prausnitzii* is an important member of the Firmicutes phylum that produces short-chain fatty acids (SCFAs), primarily through the fermentation of dietary fiber. The three major SCFAs are acetate, propionate, and butyrate. Of these, butyrate is of particular interest due to its numerous beneficial metabolic effects in the human body [39,40,41]. *F. prausnitzii* and SCFA have been associated with anti-inflammatory properties, including the downregulation of interleukin (IL)-12 and interferon (IFN)-gamma and the improvement of gut and skin barrier function [42,43]. Since the introduction of the gut–skin axis concept, there has been growing interest in the association between SCFA and inflammatory skin diseases. A reduced abundance of *F. prausnitzii* has been described in subjects with AD and psoriasis [44,45]. Koga et al. presented intriguing data demonstrating an increase in *F. prausnitzii* abundance in children with AD following oral supplementation with Kestose, a fructooligosaccharide and suggested a possible association with an improvement in AD symptoms [46]. A reduction in *F. prausnitzii* has also been identified as a distinct feature in individuals with IBD [47,48,49], and monitoring *F. prausnitzii* may even serve as a biomarker to aid in the diagnosis of intestinal disease [50,51]. Notably, a decreased abundance of *F. prausnitzii*, as observed here in the rosacea patients, has not been previously reported in rosacea patients; however, there could be a possible association with cutaneous inflammation and GI comorbidities in such patients. As this bacterium is extremely oxygen-sensitive, limiting industrial application as a probiotic, future studies are needed to investigate whether the use of a prebiotic may also increase its abundance in the gut to achieve anti-inflammatory effects in rosacea patients, as indicated in previous trials [52].

Recent research has shown that individuals with rosacea were able to identify dietary triggers more easily than beneficial foods [53]. Supporting previous data, alcohol, spices, sugar, and fried foods were perceived as leading dietary triggers, and the foods most frequently mentioned as favorable in the present cohort were high in dietary fiber, including vegetables, fruits, whole grains, and beans. These foods are known to improve gut microbiome diversity and may explain the observed subjective improvements [24,54]. However, no studies have yet been conducted to investigate the connection between dietary patterns in rosacea patients and their impact on the gut microbiome. According to a metagenomic analysis of 1135 healthy participants from a Dutch population, the Shannon diversity index decreased based on carbohydrate intake levels, followed by sugar-sweetened beverages, bread, beer, and savory snacks. Conversely, microbial diversity increased with fruit, coffee, and vegetables [55]. Due to the small sample size in the pilot study, no significant correlations were found between dietary patterns and microbiota characteristics, suggesting the need for larger studies in the future. In particular, it would be interesting to see how measures of alpha and beta diversity change with increased consumption of probiotic and prebiotic foods and whether the clinical appearance of rosacea can be positively influenced.

This pilot study has several limitations. The diversity of the gut microbiome is influenced by several factors, including culture and country. Therefore, the results of the present Western cohort in a cross-sectional study design may not be generalizable to a global population of rosacea patients. Conducting a multicenter prospective study with gut microbiome profiling would provide more insight on this matter. Clinical data from the control group were limited, which limited the comparative study of cofactors. Also, no significant associations were found between the clinical severity of rosacea or the presence of GI symptoms and microbiota characteristics, possibly due to the small sample size. However, as this was a pilot study, future studies are needed to investigate these aspects, which were beyond the scope of this study. For example, as GI symptoms were seen in more than half of the rosacea patients, they could be a possible clinical link to an altered microbiota. Future studies should therefore specifically compare the gut microbiota in rosacea patients with and without GI symptoms. A subgroup analysis of patients with different clinical severity would also be desirable to gain a deeper understanding of the clinical presentation and characteristics of the gut microbiota. Further analysis investigating the relationship between the microbiota and patients’ quality of life is also needed.

Despite these limitations, our study is significant in its presentation of significant alterations in the gut microbiota in rosacea patients compared to healthy controls. It incorporates the latest clinical rosacea classification and advanced technology and provides significant findings in the largest cohort studied on this topic. Further investigation in larger study groups and clinical trials utilizing oral probiotics and examining the gut and skin microbiome are essential to validate and expand upon these discoveries.

## 5. Conclusions

The rosacea patients showed significant changes in the gut microbiota compared to the healthy controls. This pilot study was the first to describe changes in the enterotypes and F/B ratio, with enterotype II clusters being the least common in the rosacea patients. The rosacea cohort showed reduced microbial diversity, as indicated by two alpha diversity measures, and significant dissimilarities, as indicated by the beta diversity measures, with 14 significantly different taxa compared to the healthy controls. Specifically, a reduced abundance of anti-inflammatory SCFA-producing bacteria, primarily *Faecalibacterium prausnitzii*, was found in the individuals with rosacea. While these preliminary findings need to be investigated in larger studies, they may provide a possible link with facial inflammation and the commonly reported GI symptoms in rosacea patients. In addition, the results of the present study will help to interpret data from future studies when increasing patients’ alpha and beta diversity measures through targeted dietary interventions, including oral probiotics.

## Figures and Tables

**Table 1:** Rosacea patients’ characteristics given in numbers (*n*) and respective % in relation to the total cohort, except when stated otherwise. (DLQI = Dermatology Life Quality Index, *n* = number, SD = standard deviation, ROSCO = Rosacea consensus panel).

	Rosacea Cohort (*n* = 54)	Mild (*n* = 21)	Moderate/Severe (*n* = 33)	*p*-Value
**Gender**				0.541
Female	39 (72.2)	14 (25.9)	25 (46.3)	
Male	15 (27.8)	7 (13.0)	8 (14.8)	
Age (mean ± SD, years)	45.3 (±13.6)	41.8 (±15.3)	47.5 (±12.1)	0.158
ROSCO, diagnostic and major features				
Persistent centrofacial erythema	47 (87.0)	17 (31.5)	30 (55.6)	0.411
Phymatous changes	18 (33.3)	3 (5.6)	15 (27.8)	0.021
Teleangiectasia	46 (85.2)	19 (35.2)	27 (50.0)	0.461
Papules/Pustules	43 (79.6)	14 (25.9)	29 (53.7)	0.085
Flushing	41 (75.9)	19 (35.2)	32 (59.3)	0.553
ROSCO, minor features				
Dry sensation	38 (70.4)	14 (25.9)	24 (44.4)	0.762
Burning sensation	31 (57.4)	9 (16.7)	22 (40.7)	0.100
Oedema	23 (42.6)	7 (13.0)	16 (29.6)	0.398
Stinging sensation	16 (29.6)	3 (5.6)	13 (24.1)	0.068
Rosacea persistence (mean ± SD, years)	9.1 (±8.1)	7.4 (±5.8)	10.2 (±9.2)	0.176
Current topical prescription treatment	43 (79.6)	15 (27.8)	28 (51.9)	0.305
Current systemic prescription treatment	13 (24.1)	1 (1.9)	12 (22.2)	0.009
Supplement intake (daily)	31 (57.41)	10 (18.5)	21 (38.9)	0.273
Vitamin D	24 (44.44)	8 (14.8)	16 (29.6)	0.577
Vitamin B12	12 (22.22)	7 (13.0)	5 (9.26)	0.180
Zinc	12 (22.22)	3 (5.6)	9 (16.7)	0.329
Iron	5 (9.26)	4 (7.4)	1 (1.9)	0.069
Omega-3	5 (9.26)	1 (1.9)	4 (7.4)	0.638
Others (Magnesium, iodine, selenium, folic acid)	5 (9.26)	1 (1.9)	4 (7.4)	0.638
Gastrointestinal symptoms (>3×/week)	29 (53.7)	11 (20.4)	18 (33.3)	1.000
Flatulence	19 (35.2)	8 (14.8)	11 (20.4)	0.775
Constipation	11 (20.4)	3 (5.6)	8 (14.8)	0.498
Abdominal pain	7 (13.0)	1 (1.9)	6 (11.1)	0.227
Diarrhea	4 (7.4)	1 (1.9)	3 (5.6)	1.000
Nausea	3 (5.6)	0 (0)	3 (5.6)	0.274
Irritable bowel syndrome	5 (9.3)	2 (3.7)	3 (5.6)	1.000
Chronic inflammatory bowel disease	1 (1.9)	0 (0)	1 (1.9)	1.000
Type I Allergy				
Food	9 (16.7)	2 (3.7)	7 (13.0)	0.456
Medication	4 (7.4)	3 (5.6)	1 (1.9)	0.287
Environmental (pollen, dust mites, animal hair)	16 (29.6)	4 (7.4)	12 (22.2)	0.229
Nicotine (daily)	4 (7.4)	0 (0)	4 (7.4)	0.148
Alcohol (>3×/week)	5 (9.3)	2 (3.7)	3 (5.6)	1.000
Residence	(*n* = 51)		(*n* = 30)	
Urban	45 (88.2)	19 (37.3)	26 (51.0)	1.000
Rural	6 (11.8)	2 (3.9)	4 (7.8)	1.000
Occupation	(*n* = 52)		(*n* = 31)	
Full-time job	41 (78.9)	17 (32.7)	24 (46.2)	1.000
Student/Unemployed/Retired	11 (21.2)	4 (7.7)	7 (13.5)	1.000
Stress level (±SD)	(*n* = 53)		(*n* = 32)	0.073
	4.6/10 (±2.0)	4.0/10 (±1.9)	5.0/10 (±2.0)	
Daily hours of sleep				
<7	23 (42.6)	6 (11.1)	17 (31.5)	0.158
>7	31 (58.5)	15 (27.8)	16 (29.6)	
Daily physical activity	36 (66.7%)	17 (31.5)	19 (35.2)	0.138
DLQI, mean (±SD)	5.9 (±5.7)	3.9 (±5.2)	7.1 (±5.8)	0.041

## Data Availability

The data presented in this study are available on request from the corresponding author.
